# Efficacy of Smartphone Active and Passive Virtual Reality Distraction vs Standard Care on Burn Pain Among Pediatric Patients: A Randomized Clinical Trial

**DOI:** 10.1001/jamanetworkopen.2021.12082

**Published:** 2021-06-21

**Authors:** Henry Xiang, Jiabin Shen, Krista K. Wheeler, Jeremy Patterson, Kimberly Lever, Megan Armstrong, Junxin Shi, Rajan K. Thakkar, Jonathan I. Groner, Dana Noffsinger, Sheila A. Giles, Renata B. Fabia

**Affiliations:** 1 Center for Pediatric Trauma Research, Nationwide Children’s Hospital, Columbus, Ohio; 2 Center for Injury Research and Policy, Nationwide Children’s Hospital, Columbus, Ohio; 3 Department of Pediatrics, The Ohio State University, Columbus; 4 Department of Psychology, University of Massachusetts, Lowell; 5 Research Information Solutions and Innovation, Nationwide Children’s Hospital, Columbus, Ohio; 6 The Ohio State University, Columbus; 7 Department of Pediatric Surgery, Nationwide Children’s Hospital, Columbus, Ohio; 8 Department of Surgery, The Ohio State University, Columbus

## Abstract

**Importance:**

It is unknown whether smartphone-based virtual reality (VR) games are effective in reducing pain among pediatric patients in real-world burn clinics.

**Objective:**

To evaluate the efficacy of a smartphone VR game on dressing pain among pediatric patients with burns.

**Design, Setting, and Participants:**

This randomized clinical trial included children aged 6 to 17 years who seen in the outpatient clinic of a large American Burn Association–verified pediatric burn center and level I pediatric trauma center between December 30, 2016, and January 23, 2019. Speaking English as their primary language was an inclusion criterion. Intention-to-treat data analyses were conducted from December 2019 to March 2020.

**Interventions:**

Active VR participants played a VR game; passive VR participants were immersed in the same VR environment without interactions. Both groups were compared with a standard care group. One researcher administered VR and observed pain while another researcher administered a posttrial survey that measured the child’s perceived pain and VR experience. Nurses were asked to report the clinical utility.

**Main Outcomes and Measures:**

Patients self-reported pain using a visual analog scale (VAS; range, 0-100). A researcher observed patient pain based on the Face, Legs, Activity, Cry, and Consolability–Revised (FLACC-R) scale. Nurses were asked to report VR helpfulness (range, 0-100; higher scores indicate more helpful) and ease of use (range, 0-100; higher scores indicate easier to use).

**Results:**

A total of 90 children (45 [50%] girls, mean age, 11.3 years [95% CI, 10.6-12.0 years]; 51 [57%] White children) participated. Most children had second-degree burns (81 [90%]). Participants in the active VR group had significantly lower reported overall pain (VAS score, 24.9 [95% CI, 12.2-37.6]) compared with participants in the standard care control group (VAS score, 47.1 [95% CI, 32.1-62.2]; *P* = .02). The active VR group also had a lower worst pain score (VAS score, 27.4 [95% CI, 14.7-40.1]) than both the passive VR group (VAS score, 47.9 [95% CI, 31.8-63.9]; *P* = .04) and the standard care group (VAS score, 48.8 [95% CI, 31.1-64.4]; *P* = .03). Simulator sickness scores (range, 0-60; lower scores indicate less sickness) were similar for active VR (19.3 [95% CI, 17.5-21.1]) and passive VR groups (19.5 [95% CI, 17.6-21.5]). Nurses also reported that the VR games could be easily implemented in clinics (helpfulness, active VR: 84.2; 95% CI, 74.5-93.8; passive VR: 76.9; 95% CI, 65.2-88.7; ease of use, active VR: 94.8, 95% CI, 91.8-97.8; passive VR: 96.0, 95% CI, 92.9-99.1).

**Conclusions and Relevance:**

In this study, a smartphone VR game was effective in reducing patient self-reported pain during burn dressing changes, suggesting that VR may be an effective method for managing pediatric burn pain.

**Trial Registration:**

ClinicalTrials.gov Identifier: NCT04544631

## Key Points

**Question:**

Are active smartphone virtual reality (VR) games more effective than passive VR games or standard care for pain management during pediatric burn wound care?

**Findings:**

In this randomized clinical trial that included 90 pediatric patients with burns, participants in the active VR group had significantly lower scores for overall pain compared with participants in the standard care group and for worst pain compared with participants in the passive VR group and the control group.

**Meaning:**

These findings suggest that smartphone-based VR games could be an effective pain management tool for pediatric patients with burns.

## Teaser

This randomized clinical trial evaluates the efficacy of a smartphone VR game vs standard care on dressing pain among pediatric patients with burns.

## Introduction

The American Burn Association (ABA) estimates that 250 000 US children experience burn injuries, and 6% to 8% require hospitalization annually.^1^ Dressing changes could cause severe pain, often warranting high-dose opioids.^2,3,4,5^ Acute pain is pervasive after superficial and deep partial thickness burn and during medical treatment.^6^ The most acute pain is the inflammatory nociceptive pain attributed to burned human tissue. Nociceptive pain is often followed by, and potentially exacerbated by, procedural pain associated with burn care.^7^ Burn dressing changes can cause pain that is equivalent to or worse than the initial burn injury pain.^7^

An equally important challenge is coexisting anxiety. Faucher et al^8^ hypothesized that anticipation of pain exacerbates patient anxiety, which in turn can exacerbate the pain. Prior research suggests that anxiety is particularly important during the first dressing change, as anxiety may increase over time and lead to long-term pain management challenges.^9,10^ Anxiety during burn dressing has been compared with inescapable shock or learned helplessness in pediatric patients.^11,12^

Clinical burn care often uses high-dose opioids.^1^ While opioids are effective in managing pain, they are associated with major adverse effects.^13,14^ Serious concerns have also been raised by experts about the combination use of opioids and benzodiazepine in pain management.^15,16^ In recent years, nonpharmacologic approaches have been used as adjunctive aids for acute pain management.^17,18^ Nonpharmacologic techniques, such as cognitive behavioral therapy, toy distraction, relaxation techniques, virtual reality (VR), and hypnosis, have been reported with varying degrees of success.^1^ There is an increasing body of evidence that VR is effective for pain management.^18,19^ A recent systematic review of randomized clinical trials (RCTs) that examined VR pain management between 2005 and 2015 concluded that VR games have been shown to be efficacious, easy to use, and safe, with high patient satisfaction.^18^ Furthermore, adverse effects from VR are seldom reported in pediatric patients.^18^ However, prior studies of VR among patients with burns were small, varied in terms of quality, often used computer-based VR games, or were conducted in a laboratory among healthy participants.^18^ Thus, more research is needed to prove VR clinical efficacy.^17,18^ Previous studies showed that active VR gaming provides a superior analgesic effect than passive VR for laboratory-based cold pressor pain in healthy children.^20^ However, this superiority has not been validated in actual burn clinics.

This study evaluated the feasibility, user experience, and efficacy of active vs passive smartphone VR games for pediatric burn pain management. Our central hypothesis was that active VR games could significantly better reduce pain compared with passive VR games and standard care groups.

## Methods

This RCT was designed to evaluate a smartphone VR pain alleviation tool (VR-PAT) in reducing perceived pain and observed pain during pediatric burn dressing. The study protocol appears in Supplement 1. Pediatric patients with burn injuries were recruited between December 2016 and January 2019 from an ABA-verified US pediatric burn center and randomly assigned to an active VR group, passive VR group, or a standard care control group, which used conventional distraction methods. Eligible participants were children aged 6 to 17 years (inclusive) with burn injury who were seen in the outpatient clinic and spoke English as their primary language. Patients were excluded if they had (1) a severe burn on the face or head that prevented VR use; (2) cognitive or motor impairment that prevented administration of study measures; (3) visual or hearing impairments that prevented VR interaction; or (4) did not have a legal guardian present to give consent.

A sample size and power analysis were conducted to determine the sample size required. We assumed a medium effect size (*f*^2^ = 0.15) of the active VR. Using 2 tails and alpha = .05, a fixed-effect linear regression model would offer power greater than 0.80 with a total sample size of 69 children. To ensure adequate power, we planned to recruit 30 participants for each group. The institutional review board of Nationwide Children’s Hospital reviewed and approved this study. Written informed consent (and assent for children aged 9 years and older) was collected. This study followed the Consolidated Standards of Reporting Trials (CONSORT) reporting guideline. This was a pilot project funded by intramural support, and it met the nonapplicable study definition of the Food and Drug Administration Amendments Act §801 for registration at ClinicalTrials.gov. However, a late voluntary registration was made.

**Figure 1.**
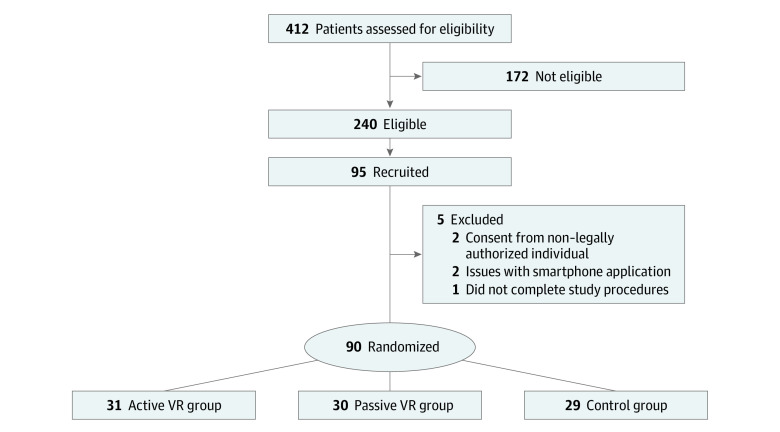
Screening and Recruitment Diagram

A total of 412 patients were consecutively screened for eligibility with pauses in enrollment due to staff availability, and 240 were deemed potentially eligible. Overall, 95 participants were ultimately recruited. Five participants were excluded because of consent from non–legally authorized member (n = 2), issues with the smartphone application (n = 2), and not completing the study procedures (Figure 1). Of the 90 participants, 31 (34%) were assigned to the active VR-PAT, 30 (33%) to the passive VR-PAT, and 29 (32%) to the control group.

### Study Procedures

Potential participants were identified via medical record review, and eligible participants were approached by a trained researcher in the outpatient clinic prior to their appointment. Following informed consent, participants were asked to fill out a preintervention survey about their demographic characteristics, pain expectation, anxiety, and pain medication use prior to the burn care. However, details about name, type, and dose of pain medication were not collected. Participants were then randomized using a 1:1:1 allocation ratio, which was balanced for sex and hosted on a Research Electronic Data Capture (REDCap) site.^21,22^ Researchers and nurses did not know the group assignment until immediately prior to intervention. One researcher implemented randomization, administered the VR-PAT (if applicable), and observed and documented pain using the Faces, Legs, Activity, Cry, and Consolability–Revised (FLACC-R) scale. After the dressing change, a second researcher, who was masked to group assignment, administered a postintervention survey about child and guardian perceived pain and subjective VR experience. Nurses were asked to report VR-PAT clinical utility. They were provided scoring on questions of helpfulness (range, 0-100; higher scores indicate more helpful) and ease of use (range, 0-100; higher scores indicate easier to use).

### Interventions

#### VR-PAT

VR-PAT was administered using lightweight, low-cost VR paired with an Apple iPhone 6 and detachable earphones (eFigure in Supplement 2). VR-PAT is a stand-alone game that was developed by the Research Information Solutions and Innovation department of Nationwide Children’s Hospital.

#### Active VR-PAT Group

Active VR participants played a VR game titled Virtual River Cruise. In this game, an otter floated a boat down a river, and players activated snow-blowing statues. The statues would emit snow if they were correctly aimed at by the child, and a thermometer placed on the boat showed decreased temperatures as more snowflakes were blown. A scoreboard placed beside the thermometer would show children the number of statues they had activated to reinforce engagement. Additionally, as the temperature dropped, snow would start piling up on the boat and its surroundings, providing a potential cooling experience for the participant. Children played the VR game by tilting their head, minimizing interference with the dressing change.

#### Passive VR-PAT Group

The passive VR participants were immersed in the same VR environment as the active VR group. However, they did not interact with the VR game.

#### Control Group

The standard care participants received routinely used distraction tools provided in the clinical setting. These included iPads, music, books, and/or talking.

### Study Outcomes and Confounding Variables

#### Primary Outcome

Patient-perceived pain, rated on a visual analog scale (VAS; range, 0-100, with higher scores indicating more pain), was the primary outcome. Self-reported time thinking about pain using VAS on an iPad and researcher-observed patient pain using FLACC-R were secondary outcomes.

#### Exploratory Outcomes

Following the burn dressing, participants and their guardians were asked to report the VR experience. Patients were asked to report adverse effect symptoms using the Simulator Sickness Questionnaire (SSQ; range, 0-60; lower scores indicate less sickness).^23^ Additionally, we collected nurse-reported utility of VR-PAT in the clinical setting, using the previously described scales.

#### Confounding Variables

Potential confounding variables included: (1) anxiety-prone traits, measured using an abbreviated State-Trait Anxiety Inventory for Children (STAI-CH)^24^; (2) expectations of VR distraction effectiveness; (3) pain medication use within 6 hours prior to the dressing change; (4) burn severity (first, second, and third degree) and percentage total body surface area (TBSA) burned. Demographic variables, such as race/ethnicity, and burn injury data were abstracted, as reported, in electronic medical records. Due to small numbers, race/ethnicity was grouped into White vs other, which included the categories of Black, Hispanic, Asian, Native American, and mixed race, in the analysis.

### Statistical Analysis

Demographic and burn characteristics were described using frequencies and percentages for the categorical variables and means and 95% CI for continuous variables. The primary outcome was compared pairwise between the 3 study groups using the grouped *t* test. A subgroup analysis was conducted regarding patient pain medication use within 6 hours prior to the burn dressing as well as by race/ethnicity.

Although randomization created comparable patients for the 3 groups, we conducted univariate and multivariate linear regression analyses to assess the impact of potential risk factors on observed pain, self-reported overall pain, and worst pain. Separate subgroup multivariate linear regression models were conducted for patients who used and did not use pain medications within 6 hours prior to the burn dressing. Statistical significance was set at α < .05, and all tests were 2-tailed. Data analyses were conducted in SAS version 9.4 (SAS Institute).

## Results

Patient demographic and burn characteristics are presented in Table 1, and baseline comparisons are shown in Table 2. Overall, the mean age of the 90 children was 11.3 years (95% CI, 10.6-12.0 years), 45 (50%) were female patients, and 51 (57%) were White patients. Most children had second-degree burns (81 [90%]). There were no differences among the comparison groups in sex distribution, mean age, percentage TBSA burned, anxiety scores, or child and caregiver VR expectation scores. Median days since the injury were also similar across the 3 groups. Of the 90 participants, 30 (33%) were given a pain medication within 6 hours prior to the burn dressing appointment, and prior pain medication use was unknown for 3 patients (3%). There was no difference between the 3 comparison groups with regard to pain medication use, although details regarding name, type, and dose of pain medication were not collected. Therefore, patients in the 3 comparison groups were comparable in terms of potential factors that could impact their pain scores.

**Table 1.** Demographic and Burn Characteristics of Study Participants

Characteristic	No. (%)			
	Distraction type			Total (N = 90)
	Active VR (n = 31)	Passive VR (n = 30)	Control (n = 29)	
Sex				
Male	16 (52)	16 (53)	13 (45)	45 (50)
Female	15 (48)	14 (47)	16 (55)	45 (50)
Age, y^a^				
6-9	10 (32)	11 (36)	16 (55)	37 (41)
10-14	13 (42)	17 (57)	8 (28)	38 (42)
15-17	8 (26)	2 (7)	5 (17)	15 (17)
Race/ethnicity				
White	23 (74)	13 (43)	15 (52)	51 (57)
Black	3 (10)	14 (47)	10 (35)	27 (30)
Hispanic	0	1 (3)	1 (3)	2 (2)
Other^b^	5 (16)	2 (7)	3 (10)	10 (11)
TBSA burned, %^a^				
Missing	0	1 (3)	2 (7)	3 (3)
<1.0	10 (32)	13 (43)	8 (28)	31 (34)
1.0-4.9	19 (61)	12 (40)	15 (52)	46 (51)
5.0-25.0	2 (7)	4 (13)	4 (14)	10 (11)
Burn degree				
Missing	1 (3)	0	1 (3)	2 (2)
First	0	0	1 (3)	1 (1)
Second	29 (94)	27 (90)	25 (86)	81 (90)
Third	1 (3)	3 (10)	2 (7)	6 (7)
Pain medication within 6 h prior to burn dressing change^c^				
Missing	1 (3)	1 (3)	1 (4)	3 (3)
No	19 (61)	19 (63)	19 (65)	57 (63)
Yes	11 (36)	10 (33)	9 (31)	30 (33)

Abbreviations: TBSA, total body surface area; VR, virtual reality.

^a^ See Table 2 for comparisons in the mean age and mean TBSA burned.

^b^ Includes Asian and Native American individuals and individuals reporting mixed race.

^c^ Details about name, type, and dose of pain medication were not collected in the survey.

**Table 2.** Comparisons by Mean Age, TBSA Burned, Anxiety Score, Child and Caregiver Expectations, and Median Days Since Injury Among Pediatric Patients With Burns

Characteristic	Mean (95% CI)			
	Overall (N = 90)	Distraction type		
		Active VR (n = 31)	Passive VR (n = 30)	Control (n = 29)
Age, y	11.3 (10.6-12.0)	12.0 (10.7-13.3)	11.3 (10.2-12.3)	10.4 (9.1-11.8)
TBSA burned, %	2.6 (1.8-3.4)	2.0 (1.1-2.8)	3.0 (1.1-4.9)	2.9 (1.5-4.3)
Abbreviated STAQI-CH-score	12.1 (11.4-12.7)	11.7 (10.7-12.6)	12.2 (11.0-13.4)	12.3 (10.9-13.7)
Child expectation				
Of fun	87.0 (83.0-91.1)	85.1 (77.0-93-3)	85.5 (78.0-92.9)	90.7 (84.7-96.8)
Of helpfulness	65.7 (59.7-71.7)	61.6 (51.2-72.0)	71.6 (62.7-80.4)	64.0 (51.2-76.8)
Caregiver expectation				
Of fun	89.1 (85.0-93.2)	90.5 (84.2-96.9)	86.8 (78.8-94.8)	89.9 (82.0-97.8)
Of helpfulness	76.4 (72.1-80.7)	75.8 (69.5-82.1)	76.3 (68.0-84.6)	77.1 (68.6-85.7)
Time since burn injury, median (IQR), d	6 (4-10)	6 (4-11)	5 (3-10)	8 (4-10)

Abbreviations: IQR, interquartile range; STAQI-CH, State-Trait Anxiety Inventory for Children; TBSA, total body surface area; VR, virtual reality.

### Primary Outcome

**Figure 2.**
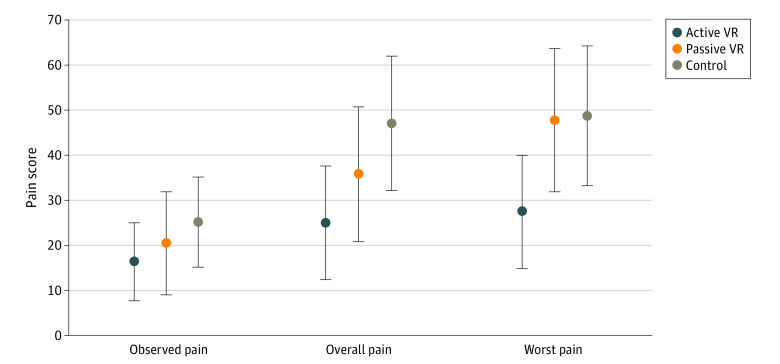
Comparison of Observed Pain, Self-reported Overall Pain, and Self-reported Worst Pain Among All Participants Pain scores range from 0 to 100, with higher scores indicating more pain. Whiskers represent 95% CIs.

Active VR participants had significantly lower reported overall pain (VAS score, 24.9; 95% CI, 12.2-37.6) compared with the standard care participants (VAS score, 47.1; 95% CI, 32.1-62.2; *P* = .02). The active VR group also had lower worst pain (VAS score, 27.4; 95% CI, 14.7-40.1) than both the standard care group (VAS score, 48.8; 95% CI, 31.1-64.4; *P* = .03) and the passive VR group (VAS score, 47.9; 95% CI, 31.8-63.9; *P* = .04) (Figure 2).

**Figure 3.**
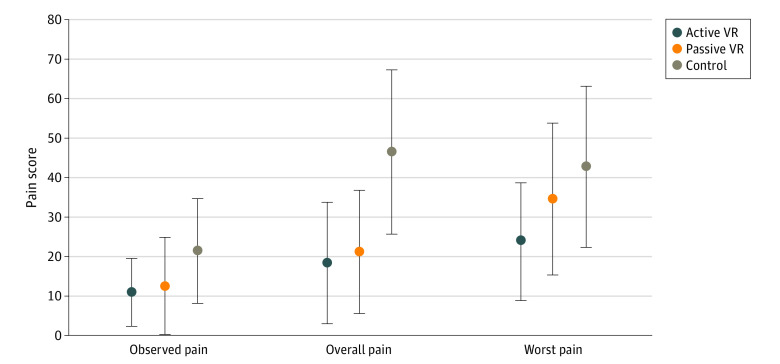
Comparison of Observed Pain, Self-reported Overall Pain, and Self-reported Worst Pain Among Participants Who Did Not Take Pain Medications Within 6 Hours of Dressing Change Pain scores range from 0 to 100, with higher scores indicating more pain. Whiskers represent 95% CIs.

When we limited the analysis to the 57 participants who were not given pain medication within 6 hours, differences were seen in both active VR and passive VR groups compared with standard care group (Figure 3). Compared with the standard care group (VAS score, 46.6; 95% CI, 25.7-67.5), self-reported overall pain score was significantly lower for both the active VR group (VAS score, 18.4; 95% CI, 2.8-34.0; *P* = .03) and the passive VR group (VAS score, 21.3; 95% CI, 5.7-36.9; *P* = .04).

Our subgroup analysis by distraction type and patient race found no significant difference in the observed and self-reported pain scores between racial and ethnic subgroups (eTable 1 in Supplement 2). We also conducted univariate and multivariate regression analyses and checked interaction terms of group assignment × age, group assignment × anxiety score, and group assignment × expectations of VR. None of the interaction terms were significant; therefore, they were not included in the final multivariate regression results (eTable 2, eTable 3, and eTable 4 in Supplement 2). In the univariate regression analysis, pain medication within 6 hours had a significant impact on self-reported overall pain (β = 20.56; 95% CI, 3.65 to 37.47; *P* = .02) and worst pain (β = 20.67; 95% CI, 2.95 to 38.38; *P* = .02). Anxiety scores were also a marginally significant factor for self-reported overall pain (β = 3.00; 95% CI, 0.45 to 5.56; *P* = .02). The higher the anxiety score, the higher the observed pain and self-reported overall pain score. In comparison with standard care, active VR had significant impact on self-reported overall pain (β = −22.20; 95% CI, −41.80 to −2.60; *P* = .03) as well as worst pain (β = −21.34; 95% CI, −41.69 to −0.98; *P* = .04). Other significant factors were age, percentage TBSA, burn degree, and child expectation of VR. Multivariate regression results in eTable 3 and eTable 4 in Supplement 2 show that most significant variables in univariate regression became nonsignificant, with exception of race (White patients: β = −22.05; 95% CI, −41.12 to −2.97; *P* = .02), active VR (β = −26.39; 95% CI, −50.12 to −2.67; *P* = .03), and passive VR (β = −24.09; 95% CI, −47.11 to −1.09; *P* = .04) on self-reported overall pain among patients who did not use pain medication within 6 hours (eTable 3 in Supplement 2).

### Other Outcomes

Most children and caregivers reported satisfaction with VR-PAT (eTable 5 and eTable 6 in Supplement 2). While not statistically different, the proportion of time thinking about pain during the dressing change (measured on 100-point VAS, with higher scores indicating more time thinking about pain) was the least for participants in the active VR group (mean, 20.3; 95% CI, 8.9-31.8) vs those in the passive VR group (mean, 34.6; 95% CI, 20.2-49.0) and in the standard care group (mean, 36.5; 95% CI, 20.8-52.2). Clinicians also gave the VR-PAT tool high utility scores in terms of helpfulness (active VR: 84.2; 95% CI, 74.5-93.8; passive VR: 76.9; 95% CI, 65.2-88.7) and ease of use (active VR: 94.8, 95% CI, 91.8-97.8; passive VR: 96.0, 95% CI, 92.9-99.1). Mean SSQ scores were similar for the active VR group (19.3; 95% CI, 17.5-21.1) and passive VR group (19.5; 95% CI, 17.6-21.5).

## Discussion

The smartphone VR-PAT showed efficacy in reducing observed and patient self-reported pain during burn dressing changes. Patients and their caregivers were satisfied with the VR-PAT, and children considered the VR-PAT fun, engaging, and realistic. Nurses also reported that the smartphone VR was easy to use in the clinical setting. Child age, burn degree, anxiety, pain medication use within 6 hours, and expectation of VR fun impacted patient self-reported overall pain.

Researchers have made strides in the past 2 decades in showing the efficacy of VR games as an adjunctive nonpharmacologic pain management approach during burn dressing.^25,26,27^ Prior studies reported that immersive VR games could provide clinically meaningful pain relief (30%-50% reductions in subjective pain scores) compared with standard care in pediatric burn dressing procedures.^28,29,30^ Our study added to the evidence supporting VR as an effective pediatric burn pain management tool. Furthermore, this study contributes to the growing body of evidence that active VR games are superior to passive VR games. Researchers conducted laboratory-based cold pressor trial VR studies among healthy children and young adults and reported that active VR was more effective than passive VR games.^31,32,33^ Loreto-Quijada et al^34^ reported that VR had a greater effect on the cognitive capacity of healthy children during the cold pressor experiment compared with the control group. Our study provided evidence that active VR games are more effective than passive VR games in reducing pediatric pain during clinical burn care.^29,30^

According to the cognitive-affective model of pain,^35^ active VR that requires engagement would be more efficacious than passive VR viewing, as the active VR would pose a heavier attention load on a person’s cognitive system. Although VR games have been widely evaluated as a pain management tool in children as well as in adults, only a few studies have attempted to directly test the hypothesis that active VR games are superior to passive VR.^20,33^ Moreover, clinical implications that could be drawn from prior studies that compared active vs passive VR games are limited, as almost all studies were conducted in laboratory-based cold pressor trials of healthy individuals.^20,33^ One clinical study by Jeffs et al^29^ compared a passive task of watching a movie to playing a VR game on a computer and reported that the active VR group self-reported less pain during wound care than either the passive movie watching or standard care group. Although those findings were consistent with the cognitive-affective model of pain,^35^ it is also possible that the superior effect of the active VR was solely due to the fact that the active VR was more fun rather than the fact that it activated central attentional processing. Furthermore, their passive and active VR game environments were different. Our study attempted to address this limitation by using the same VR environment; therefore, our findings provide stronger evidence, closer to isolating the benefits of active engagement via active VR for pain reduction. The potential cooling effect of the snow environment in both the active and passive VR might also have some effect on reducing self-reported pain in comparison with standard care groups.^29^

A 2016 large survey among 378 nurses and physicians at 133 ABA-verified burn centers^1^ reported that oxycodone and morphine or fentanyl were the most frequently used opioid medications for dressing changes. An RCT study of adolescents (aged 11-17 years) reported that participants using off-the-shelf VR received significantly fewer rescue doses of a nitrous oxide/oxygen mixture compared with the standard care group.^36^ Researchers strongly advocate for more funding to support pain research of nonpharmacologic approaches, such as VR, with a hope that such efforts would make a significant contribution to reducing opioid medications during painful medical procedures.^37^ In our study, although none of the patients needed rescue opioids, pain medication use within 6 hours prior to burn dressing was a significant factor for both observed and self-reported pain. Future VR study needs to focus on reducing opioids as a primary outcome.

One advantage is our VR game could be played using a smartphone, making it easy to set up and therefore accessible. Pediatric patients and their caregivers were very satisfied with the VR-PAT and reported that the VR-PAT was fun, engaging, and realistic. Nurses also reported that the VR-PAT could be easily implemented in their clinical care. VR systems that need large equipment space (eg, a computer) and training of clinical staff are generally not well accepted by clinics.^38,39^

### Limitations

This pilot study has a few limitations. First, our findings were only generalizable to outpatient burn clinics where actual burn dressing changes are generally short (<30 minutes). The second limitation is that this pilot study did not test the efficacy of VR-PAT in repeated burn dressing changes. Prior small studies reported that VR games could still be effective in reducing pain over multiple repeated burn dressing changes among pediatric and adult patients with burns.^40,41^ The third limitation is the potential bias in the researcher-observed pain. Due to the study procedures and patient VR interaction, it was almost impossible to totally mask the observer to avoid the bias. The fourth limitation is lack of detail data about pain medication use within 6 hours prior to the burn dressing. Fifth, due to safety concerns and need for self-reporting of pain and experience, only pediatric patients with burns aged 6 to 17 years were included, while a national study by our team found that approximately 60% of pediatric patients with burns in the US are children aged 4 years or younger.^42^

## Conclusions

In this study, a smartphone VR game was effective in reducing self-reported pain during pediatric burn dressing changes. Nurses also reported that VR-PAT could be easily implemented in their clinical practice. Future research needs to prove the opioid-sparing effects of smartphone VR games.

## Notes

Supplement 1. Trial Protocol

Supplement 2. **eFigure 1.** Smartphone VR-PAT **eTable 1.** Subgroup Analysis: Mean Pain Score by Distraction Type and Race **eTable 2.** Univariate Linear Regression Analysis of Demographic and Burn Characteristics, Pain Medication Prior to Burn Dressing, Child Expectation of VR, and VR Group on Pain Scores, All Children **eTable 3.** Multivariate Linear Regression Analysis of Demographic and Burn Characteristics, Child Expectation of VR, and VR Group on Pain Scores Among Children Who Did Not Use Pain Medication Within 6 Hours of Burn Dressing **eTable 4.** Multivariate Linear Regression Analysis of Demographic and Burn Characteristics, Child Expectation of VR, and VR Group on Pain Scores Among Children Who Did Use Pain Medication Within 6 Hours of Burn Dressing **eTable 5.** Pediatric Patients With Burns Reported VR Experiences **eTable 6.** Secondary Outcomes of VR Game Experience of Pediatric Patients With Burns

Supplement 3. Data Sharing Statement
