# Supplementary material for: Efficacy of Smartphone Active and Passive Virtual Reality Distraction vs Standard Care on Burn Pain Among Pediatric Patients: A Randomized Clinical Trial
Source: JAMA Netw Open. 2021 Jun 21;4(6):e2112082. doi: 10.1001/jamanetworkopen.2021.12082 (PMC8218073; doi:10.1001/jamanetworkopen.2021.12082)
Supplement: Supplement 1. — Trial Protocol [file jamanetwopen-e2112082-s001.pdf]

Title: Active and Passive Virtual Reality Distraction for Pain Management during Pediatric Burn Dressing Changes

1. SPECIFIC AIMS

Burns are one of the leading causes of pediatric injuries with over 100,000 children injured every year.[1] Effective pain management during burn dressing changes is essential for pediatric patients' medical care experience and long-term psychological health.[2-5] Consistent with the Cognitive-Affective Model of Pain (CAMP) which maintains that pain interrupts and demands attention,[6] virtual reality (VR), as an emerging and promising technology, has been increasingly used as a non-pharmaceutical analgesic method for pain management during dressing changes.[7] However, existing evidence on the effectiveness of this VR-based analgesic in pediatric burns patients is mixed due to the lack of adequately powered randomized control trials. Furthermore, previous studies have showed that *active* virtual reality (VR) gaming provides a superior analgesic effect to *passive* VR experiences for laboratory-induced pain among healthy children.[8] However, this superiority of active versus passive VR distraction has not been well validated among pediatric burn patients using rigorously-designed randomized controlled trials. Additionally, clinical practitioners have long recognized the effect of pediatric patients' developmental and psychological characteristics on their coping with pain during medical procedures.[7] But to our knowledge, no published studies have investigated these important moderating effects when using VR for pediatric pain management during burn dressing changes. The <u>overall objective</u> of this proposal, which is the first step to systematically address this gap, is to evaluate the efficacy and influencing factors of an innovative VR distraction tool for pain management during pediatric burn dressing changes. Our <u>*central hypothesis*</u> is that active VR gaming platforms can provide the most significant pain remediation as a distraction tool for pain management during pediatric burn dressing changes.

<u>Aim 1</u>. Conduct a three-group randomized controlled trial to evaluate the efficacy of active VR distraction versus passive VR distraction

<u>*Hypothesis 1.1*</u>: Pediatric burn patients using the VR tool (active or passive) will have significantly lower pain compared to those using standard distraction methods during dressing changes.

<u>*Hypothesis 1.2*</u>: Pediatric burn patients using the active VR distraction will have significantly lower pain than those using the passive VR distraction during dressing changes.

<u>Aim 2</u>. Examine the moderating effect of developmental and psychological factors on the efficacy of active VR distraction.

<u>*Hypothesis 2.1*</u>: Age, personality, and expectations will moderate the efficacy of the active VR.

2. BACKGROUND AND SIGNIFICANCE

The Centers for Disease Control and Prevention (CDC) rank burn injuries as one of the leading causes of pediatric injuries.[1] In 2013 in the United States alone, over 114,000 children and adolescents less than 18 years (with most being very young) sustained nonfatal injuries.[1] Dressing changes, a critical procedure in burn wound care, were identified by pediatric patients as one of the major contributors to intense pain.[3,4] Psychologically, the pain experienced during dressing changes may add to the original burn trauma, affecting patients' long-term functioning both psychologically and behaviorally.[2,5] Therefore, pain management becomes essential for 'patient-centered' burn wound care.[9]

According to the Cognitive-Affective Model of Pain[6], the perception of pain demands cognitive attention. Thus to achieve effective non-pharmacologic pain management, the cognitive route leading to pain perception (i.e., the pain pathway) must be interrupted. One recent application of this cognitive neuroscience theory in pain management is the use of virtual reality (VR), where the immersive environment provides great potential for engaging individuals' attention-resources to interrupt the pain perception.[7,10] Despite VR's increasing clinical application in the management of chronic pain, few rigorously-designed studies evaluating the efficacy of VR for pain management during pediatric burn dressing changes have been conducted. Our literature search identified only two existing randomized trials evaluating immersive VR for pain management during pediatric burn dressing changes, however, both studies suffered from small/ limited age group samples (an average of 15 children per group; 10 years and above).[11,12] We are also aware of a randomized trial using VR distraction during pediatric burn dressing changes partially funded by NCH intramural grant in 2009, which failed to find a significant effect of VR on pain management. After in-depth discussion with the Principle Investigator of the project (Dr. Catherine Butz), we identified two critical limitations of that previous study. First, due to the technological limitations of VR at the time of study, VR equipment was both bulky and heavy, which greatly affected the utility of VR in burn dressing settings and negatively affected patients' VR experience and hence the effec-

tiveness of the distraction. The complex VR system used in the study also interfered with the recruitment and study implementation. Our study will overcome this limitation by incorporating the latest VR technology that is reliable, compact, light-weight and child-friendly. Second, multiple raters (usually nurses) were recruited to assess patients' pain intensity in the previous study. However, our recent study showed that there were considerable variations in rating pain scores for the same patient by different nurses, dependent upon nursing experience.[13]

Furthermore, according to the Cognitive-Affective Model of Pain, an "active" VR tool would surpass a "passive" VR tool in its efficacy for pain reduction, because the "interaction" between child and VR tool would pose a heavier cognitive load on the individual's attention system.[6] Although scarce evidence from laboratory-induced pain studies among healthy participants supported that "active" VR is significantly better than "passive VR" in raising individuals' pain threshold and tolerance level,[8] the superiority of an "active" versus "passive" VR tool has yet to be validated in pediatric burn-pain management during dressing changes. Our literature search only identified one randomized controlled trial (RCT) attempting to address this topic, but with a flawed design.[11] Aside from the restricted sample size (10/group) and age range (10+), this study used different media content for the active VR game and the passive VR movie. The lower pain experienced by the VR gaming group could not be differentiated from the 'interaction' element of VR game, or the media content associated with the game.

Finally, two recent systematic reviews (2010 and 2012) identified the lack of empirical evidence regarding the moderating effect of developmental and psychological factors on the effectiveness of VR distraction on pediatric procedural pain.[7,14] The existing literature has a paucity of published research addressing this important topic. From a clinical perspective, the moderating effects are significant in daily practice. Child Life Specialists are required to utilize different distraction strategies for pediatric patients with varying individual characteristics (e.g., developmental stages).[15] Providing evidence-based guidance to maximize the benefit of VR-based distraction tools for pediatric burn patients is therefore critical.[16]

The *scientific significance* of the present study is twofold. First, this project will be the first randomized controlled trial in the field evaluating the efficacy of active versus passive virtual reality distraction among a broader sample of pediatric burn patients during dressing changes. VR is a rapidly developing technology in recent years and has emerged as a promising technology in medical settings. This pilot trial will provide the empirical foundation for the Center for Pediatric Trauma Research to become a leading research center using VR for pediatric trauma patients. Second, this project will be the first study in the literature to examine the impact of child-specific moderating characteristics on VR distraction efficacy from a developmental-psychology perspective. This proposed project also holds important *clinical implications*. Findings from this study will provide empirical data to support the development of evidence-based, individualized distraction strategies to reduce pediatric burn patients' pain during dressing changes. The preliminary data from this study will be critical when applying for external funding to address this important topic in a broader range of pediatric populations and medical procedures.

3 PRELIMINARY DATA

3.1 Nationwide Children's Hospital (NCH) Burn Program

From 2011 to 2015, the Burn Clinic at Nationwide Children's Hospital (NCH) has provided comprehensive multidisciplinary care to on-average 250 outpatient burn patients (<18 years) annually, with 100 meeting criteria for this proposed project (see detailed criteria in Section 4.1). The Center for Pediatric Trauma Research has established a mutual agreement with the NCH Burn Program to carry out this pilot randomized trial at the NCH Burn Clinic. We anticipate recruitment rate of at least 90% among eligible patients over 16 months based on the experience of our clinical co-investigators. We will actively monitor the recruitment at the Burn Clinic and review the progress after the start of the study. In the meantime, we will also identify and recruit from the inpatient burn unit at the NCH Burn Program, which admits on average 150 pediatric patients every year, with 50 meeting the study criteria. The protocol feasibility and VR utility/experience will be documented and compared between the two settings to guide future extramural grant applications.

3.2 Expertise and Previous Research Experience

The proposed study will be conducted by an interdisciplinary team consisting of faculty co-investigators from the Nationwide Children's Hospital Trauma Program (Dr. Rajan Thakkar, Dr. Jonathan Groner, and Dr. Renata Fabia) and The Research Institute at Nationwide Children's Hospital (Dr. Huiyun Xiang). The expertise of this assembled team is well published with variety of original research articles,[17-24] including burn-associated hospitalizations,[23] patterns of burn injuries among children with disabilities,[24] and posttraumatic stress in parents of young children post-burn.[19] Dr. Jiabin Shen, a postdoc research fellow at the Center for Pediatric Trauma

Research, has extensive research experience in VR-based interventions[25] and randomized clinical trials[26,27] with a manuscript under review evaluating nurse accuracy in pain assessment using the Face, Legs, Activity, Crying, and Consolability (FLACC) scale.[13] Additional personnel will include Child Life Support Specialist, Lisa Kappy (with extensive experience in administrating distraction tools for pediatric burn patients).

4. RESEARCH DESIGN AND METHODS

4.1 Participants

This study will recruit 90 pediatric patients treated for burn injuries at Nationwide Children's Hospital Burn Program. Inclusion criteria are pediatric burn patients (6-17 years, inclusive) who require dressing changes and where English is the primary language of the household. Pediatric burn patient exclusion criteria include severe burn injury(s) on the face/head preventing utilization of VR, and cognitive/motor impairment preventing valid administration of study measures. A Microsoft Excel spreadsheet will be created for use by the two study recruiters in order to keep track of all patients meeting the age eligibility requirement. This eligibility and enrollment spreadsheet will include those who are not eligible (based on exclusion criteria) and eligible patients, both those who were enrolled and those who declined or were not yet approached for participation. The spreadsheet will include the following variables: any exclusion criteria met, appointment date/time, MRN, age, gender, date of burn injury, mechanism of injury, inpatient/outpatient, recruited (yes or no), and assigned study group. The rationale behind the eligibility spreadsheet is that patients have multiple follow-up appointments in the Burn Clinic. If the study recruiters have already deemed a patient eligible or ineligible, or if they have already been recruited, then the spreadsheet will be the quickest way to see that and eliminate extra screening.

4.2 Intervention

We will establish a three-group, between-subject, randomized controlled trial. Following recruitment, participants will be randomly assigned to one of three groups (30/group) prior to the dressing change procedure using a computerized randomization procedure.

Active Virtual Reality (VR) Distraction: Participants in the active VR distraction group will play an engaging and age-appropriate virtual reality game (Virtual River Cruise) in an immersive virtual reality environment. The VR environment will be created using an Apple iPhone paired with Google Cardboard, a light-weight low-cost virtual reality headwear viewer, and a wired disposable earphone. Participants will interact with the virtual environment by collecting totems along the river via slightly tilting the head, minimizing potential interference with the dressing change procedure.

Passive Virtual Reality (VR) Distraction: Participants in the passive VR distraction group will also be immersed in the same virtual reality environment as the active VR distraction group; but instead of playing the VR game, participants in this group will passively experience the river cruise without the action of totem collection.

Standard Distraction: Participants in the standard distraction group will receive routinely used distraction tools provided in the clinical setting, such as iPads, music, books, and/or talking.**4.3 Measures**

Observational, child-report, parent-report, and nurse/child-life report measures will be used in this study. Parents will be allowed to assist their child in understanding and reporting on child-report measures as needed.

a. Pain Assessment

As the primary outcome, pain will be measured using behavioral observation and patient reports.

Behavioral observation. During the training phase, two research associates will receive extensive training on pain rating and will both be designated to rate at least the first 20% of participants' pain using the Face, Legs, Activity, Crying, and Consolability revised (FLACC-r) scale on site until their inter-rater reliability reaches beyond 0.90 (using weighted kappa for ordinal data). After that point, one research associate will complete the FLACC-r rating on his/her own. The FLACC-r scale uses an easily understood 0-2 metric for assessing a child's pain in five specific domains (face, legs, activity, crying, and consolability). Inter-rater reliability and validity in procedural pain assessment have been demonstrated for children (0-18 years) among well-trained raters. The total FLACC-r scale scores range from 0 to 10, with lower scores indicating less pain.

Patient reports. The second research associate, unaware of group assignment or FLACC ratings, will survey each patient after the dressing changes using a 100 mm VAS on these three questions:

- Worst pain: "How painful would you rate your worst pain during this dressing change?"
- Average pain: "How painful would you rate your average pain for this dressing change?"

- Pain time: "How much time did you spend thinking of pain during this dressing change?"

Parent report. The second research associate will ask the parent/caregiver of each patient about their perception of child's pain intensity using a visual analog scale (VAS).

b. Anxiety-prone Personality

Prior to the dressing change, a research associate will assess the degree the patient's personality is anxiety-prone using an abbreviated version of the Trait Anxiety Subscale of the State-Trait Anxiety Inventory for Children (STAI-CH), a well-established instrument for measuring children's personality traits related to anxiety. Higher scores indicate greater proneness of personality to anxiety.

c. Expectations toward Distraction Effectiveness

Prior to the dressing change, a research associate will assess patients via a 100 mm VAS regarding their attitudes and expectations toward distraction efficacy, by asking two questions: "*How much would you like to be distracted for dressing changes?*" and "*How much do you think distraction is effective in reducing pain during dressing changes?*" Expectation scores will be the average of the two responses. Higher scores indicate higher expectation levels.

d. VR Feasibility and Experience

Observed VR experience**.** Measures recorded by the first research associate in observing the VR experience will include the following: a) the percent of time participants spent in the VR environment during the entire burn dressing-change procedure; b) the percent of participants declining to use the VR tool as distraction; and c) the number of times participants voluntarily interrupted the VR experience during the procedure.

Parent/Self-reported VR experience. Reporting to the second research associate, participants and their parent/caregiver will rate on a 100 mm VAS the following: degree of sickness and nausea, degree of realism experienced in the VR environment, degree of pleasure/fun associated with playing the VR game, degree of satisfaction with the VR experience, and perceived level of engagement with VR distraction during the procedure. In addition, the child on the two VR conditions will report on the Simulator Sickness Questionnaire developed by Kennedy and colleagues, which is a standardized easy-to-implement measurement of virtual reality-based simulator experience with well-validated reliability and internal/external validity.[28]

Utility of VR in Clinical Settings. The practicality of the VR experience as a pain management tool for pediatric burn dressing changes will be assessed by asking the Child Life Specialist or Nurse the following questions: "Is this distraction tool helpful during pediatric burn dressing changes?" and "Is this distraction tool easy to use during pediatric burn dressing changes?" Additionally, we will actively monitor the protocol feasibility in recruitment, VR implementation, and outcome assessment throughout the study.

e. Demographic and Injury Information

We will obtain date of birth, gender, ethnicity, dosage of sedatives/painkillers, medical record number, patient name, Total Body Surface Area (TBSA) and the Abbreviated Injury Score (AIS) from individual patient's medical record. Using a 4-point Likert scale, a Nurse will determine the extent of the wound (new burn, partially healed burn, mostly healed burn, completely healed).

We will use paper or electronic versions of the REDCap survey. Using the paper forms will eliminate any technology issues that do and can arise during data collection in the clinical setting. Before arriving to the clinical setting, researchers will print out the REDCap survey. Researchers will read and ask the questions to the patient, parent, and child life specialist/nurse and mark down their answers on the paper version. At the end of the session, a research associate will input the data into REDCap. A second research associate will validate the data entry and then shred the paper version. If the validation cannot occur immediately after data entry, the paper version will be stored in a locked cabinet and shredded once validated in the electronic dataset.

4.4 Data Analysis

Aim 1 will evaluate the efficacy of active VR distraction during dressing changes compared to passive VR distraction and standard distraction. First, descriptive statistical analysis will be conducted to understand the observed VR experience by nurses, self-reported VR experience by patients, and the utility of VR in clinical settings as reported by nurses. Second, to test **Hypothesis 1.1** and **1.2**, fixed-effect linear multiple regression models will be constructed and tested with pain scores (measured via observational and patient-report measures; **4.3 Measures**) as dependent variables and group assignment (i.e., active VR, passive VR, and standard distraction) as the primary predictor. Because randomization might not eliminate all pre-intervention between-group differences due to the relatively small sample size of this pilot study, we will include those

baseline variables that emerged with significant differences between groups as covariates in regression models. The multiple measures used to assess pain will be standardized and aggregated into a composite pain score if they correlate with each other; otherwise, separate analyses will be conducted for each pain measurement.

<u>Aim 2</u> is to evaluate moderating effect of psychological factors on the efficacy of active VR distraction. To test **Hypothesis 1.2**, the same multiple linear regression models as Aim 1 will be conducted with the addition of three interaction terms for detecting moderating effect: group assignment × age, group assignment × personality, and group assignment × expectations. Coefficients with $p < 0.05$ for the interaction terms will indicate significant moderating effect of respective developmental and psychological factors.

4.5 Sample Size and Power Analysis

A power analysis was conducted to determine the sample size required for the randomized controlled trial. We assumed a medium effect size ($f^2 = 0.15$) of the active VR distraction. Using 2 tails and alpha = 0.05, a fixed-effect linear multiple regression model offers power > 0.80 with 68 children for the total sample size. Our study sample (N=90) will have adequate power to detect the intervention effect.

5. EXPECTED OUTCOMES

One important outcome of the present study will be to establish the extent to which active VR distraction is superior to passive VR distraction (and standard distraction) in pain management for pediatric burn patients during dressing changes. If the superiority of active VR distraction in pain reduction during dressing changes is demonstrated, our study will provide critical preliminary data for a large-scale randomized controlled trial, as well as for other applications in procedure pain management. If the passive VR is found equally effective as the active VR and superior to the standard distraction, our study would suggest that the patient's pain could be effectively reduced by a simple/low-cost VR video with no 'interactive' burden on the patient's end. This cost effective measure would contribute to a wider adoption of this effective pain management strategy in clinical settings.

6. TIMELINE

	2016						2017												2018					
**Proposed Activity**	**7**	**8**	**9**	**10**	**11**	**12**	**1**	**2**	**3**	**4**	**5**	**6**	**7**	**8**	**9**	**10**	**11**	**12**	**1**	**2**	**3**	**4**	**5**	**6**
Protocol development and IRB approval																								
Participant recruitment and data collection																								
Data entry, cleaning, and analysis																								
Preparation and submission of 1st manuscript (Specific Aim 1)																								
Preparation and submission of 2nd manuscript (Specific Aim 2)																								

REFERENCES

1. Centers for Disease Control and Prevention, National Center for Injury Prevention and Control, WISQARS™ (Web-based Injury Statistics Query and Reporting System). http://www.cdc.gov/injury/wisqars/. Accessed January 2016.

2. Smith JS, Smith KR, Rainey SL. The psychology of burn care. *J Trauma Nurs.* Jul-Sep 2006;13(3):105-106.

3. Choiniere M, Melzack R, Rondeau J, Girard N, Paquin MJ. The pain of burns: characteristics and correlates. *J Trauma.* Nov 1989;29(11):1531-1539.

4. Solowiej K, Upton D. Painful dressing changes for chronic wounds: assessment and management. *Br J Nurs.* Nov 8-21 2012;21(20):20, 22, 24-25.

5. Das DA, Grimmer KA, Sparnon AL, McRae SE, Thomas BH. The efficacy of playing a virtual reality game in modulating pain for children with acute burn injuries: a randomized controlled trial [ISRCTN87413556]. *BMC Pediatr.* 2005;5(1):1.

6. Eccleston C, Crombez G. Pain demands attention: a cognitive-affective model of the interruptive function of pain. *Psychol Bull.* May 1999;125(3):356-366.

7. Malloy KM, Milling LS. The effectiveness of virtual reality distraction for pain reduction: a systematic review. *Clin Psychol Rev.* Dec 2010;30(8):1011-1018.

8. Dahlquist LM, McKenna KD, Jones KK, Dillinger L, Weiss KE, Ackerman CS. Active and passive distraction using a head-mounted display helmet: effects on cold pressor pain in children. *Health Psychol.* Nov 2007;26(6):794-801.

9. Hoffman HG, Doctor JN, Patterson DR, Carrougher GJ, Furness TA, 3rd. Virtual reality as an adjunctive pain control during burn wound care in adolescent patients. *Pain.* Mar 2000;85(1-2):305-309.

10. Mott J, Bucolo S, Cuttle L, et al. The efficacy of an augmented virtual reality system to alleviate pain in children undergoing burns dressing changes: a randomised controlled trial. *Burns.* Sep 2008;34(6):803-808.

11. Jeffs D, Dorman D, Brown S, et al. Effect of virtual reality on adolescent pain during burn wound care. *J Burn Care Res.* Sep-Oct 2014;35(5):395-408.

12. Kipping B, Rodger S, Miller K, Kimble RM. Virtual reality for acute pain reduction in adolescents undergoing burn wound care: a prospective randomized controlled trial. *Burns.* Aug 2012;38(5):650-657.

13. Shen J, Giles SA, Kurtovic K, et al. Evaluation of nurses' accuracy in rating procedural pain among pediatric burn patients using the Face, Legs, Activity, Cry, Consolability (FLACC) scale. *Manuscript under review.* 2016.

14. Koller D, Goldman RD. Distraction techniques for children undergoing procedures: a critical review of pediatric research. *J Pediatr Nurs.* Dec 2012;27(6):652-681.

15. Koller D. Child Life Assessment: Variables Associated with a Child's Ability to Cope with Hospitalization. Child Life Council Evidence-Based Practice Statement. Approved by the Child Life Council Executive Board. 2008. https://www.childlife.org/Resource%20Library/EBPStatements.cfm.

16. Cramer-Berness LJ, Friedman AG. Behavioral Interventions for Infant Immunizations. *Children's Health Care.* 2005/06/01 2005;34(2):95-111.

17. Askegard-Giesmann JR, Besner GE, Fabia R, Caniano DA, Preston T, Kenney BD. Extracorporeal membrane oxygenation as a lifesaving modality in the treatment of pediatric patients with burns and respiratory failure. *J Pediatr Surg.* Jun 2010;45(6):1330-1335.

18. Fabia R, Groner JI. Advances in the care of children with burns. *Adv Pediatr.* 2009;56:219-248.

19. Odar C, Kirschman KJ, Pelley TJ, Butz C, Besner GE, Fabia RB. Prevalence and correlates of posttraumatic stress in parents of young children postburn. *J Burn Care Res.* May-Jun 2013;34(3):299-306.

20. Paddock HN, Fabia R, Giles S, et al. A silver-impregnated antimicrobial dressing reduces hospital costs for pediatric burn patients. *J Pediatr Surg.* Jan 2007;42(1):211-213.

21. Pielesz A, Machnicka A, Gawlowski A, Fabia J, Sarna E, Binias W. Inhibitors of thermally induced burn incidents - characterization by microbiological procedure, electrophoresis, SEM, DSC and IR spectroscopy. *Analyst.* Jul 7 2015;140(13):4599-4607.

22. Rocourt DV, Hall M, Kenney BD, Fabia R, Groner JI, Besner GE. Respiratory failure after pediatric scald injury. *J Pediatr Surg.* Sep 2011;46(9):1753-1758.

23. Shields BJ, Comstock RD, Fernandez SA, Xiang H, Smith GA. Healthcare resource utilization and epidemiology of pediatric burn-associated hospitalizations, United States, 2000. *J Burn Care Res.* Nov-Dec 2007;28(6):811-826.

24. Chen G, Smith GA, Ranbom L, Sinclair SA, Xiang H. Incidence and pattern of burn injuries among children with disabilities. *J Trauma.* Mar 2007;62(3):682-686.

25. Schwebel DC, Shen J, McClure LA. How do children learn to cross the street? The process of pedestrian safety training. *Traffic Inj Prev.* Jan 13 2016:0.

26. Shen J, Pang S, Schwebel DC. Evaluation of a Drowning Prevention Program Based on Testimonial Videos: A Randomized Controlled Trial. *J Pediatr Psychol.* Nov 6 2015.

27. Shen J, Pang S, Schwebel DC. A Randomized Trial Evaluating Child Dog-Bite Prevention in Rural China Through Video-Based Testimonials. *Health Psychol.* Nov 2 2015.

28. Kennedy RS LN, Berbaum KS, Lilienthal MG. Simulator sickness questionnaire: An enhanced method for quantifying simulator sickness. *The international journal of aviation psychology.* 1993;1(3):203-220.
