# Supplementary material for: Efficacy of Smartphone Active and Passive Virtual Reality Distraction vs Standard Care on Burn Pain Among Pediatric Patients: A Randomized Clinical Trial
Source: JAMA Netw Open. 2021 Jun 21;4(6):e2112082. doi: 10.1001/jamanetworkopen.2021.12082 (PMC8218073; doi:10.1001/jamanetworkopen.2021.12082)
Supplement: Supplement 2. — eFigure 1. Smartphone VR-PAT eTable 1. Subgroup Analysis: Mean Pain Score by Distraction Type and Race eTable 2. Univariate Linear Regression Analysis of Demographic and Burn Characteristics, Pain Medication Prior to Burn Dressing, Child Expectation of VR, and VR Group on Pain Scores, All Children eTable 3. Multivariate Linear Regression Analysis of Demographic and Burn Characteristics, Child Expectation of VR, and VR Group on Pain Scores Among Children Who Did Not Use Pain Medication Within 6 Hours of Burn Dressing eTable 4. Multivariate Linear Regression Analysis of Demographic and Burn Characteristics, Child Expectation of VR, and VR Group on Pain Scores Among Children Who Did Use Pain Medication Within 6 Hours of Burn Dressing eTable 5. Pediatric Patients With Burns Reported VR Experiences eTable 6. Secondary Outcomes of VR Game Experience of Pediatric Patients With Burns [file jamanetwopen-e2112082-s002.pdf]

Supplemental Online Content

Xiang H, Shen J, Wheeler KK, et al. Efficacy of smartphone active and passive virtual reality distraction vs standard care on burn pain among pediatric patients: a randomized clinical trial. *JAMA Netw Open*. 2021;4(6):e2112082. doi:10.1001/jamanetworkopen.2021.12082

eFigure. Smartphone VR-PAT

eTable 1. Subgroup Analysis: Mean Pain Score by Distraction Type and Race

eTable 2. Univariate Linear Regression Analysis of Demographic and Burn Characteristics, Pain Medication Prior to Burn Dressing, Child Expectation of VR, and VR Group on Pain Scores, All Children

eTable 3. Multivariate Linear Regression Analysis of Demographic and Burn Characteristics, Child Expectation of VR, and VR Group on Pain Scores Among Children Who Did Not Use Pain Medication Within 6 Hours of Burn Dressing

eTable 4. Multivariate Linear Regression Analysis of Demographic and Burn Characteristics, Child Expectation of VR, and VR Group on Pain Scores Among Children Who Did Use Pain Medication Within 6 Hours of Burn Dressing

eTable 5. Pediatric Patients With Burns Reported VR Experiences

eTable 6. Secondary Outcomes of VR Game Experience of Pediatric Patients With Burns

This supplemental material has been provided by the authors to give readers additional information about their work.

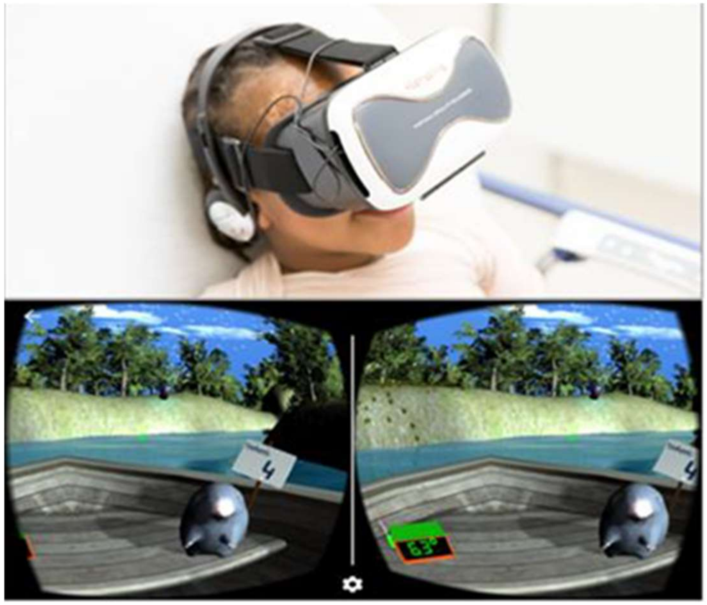

eFigure. Smartphone VR-PAT

© 2021 Xiang H et al. *JAMA Network Open.*

eTable 1. Subgroup Analysis: Mean Pain Score by Distraction Type and Race

	Distraction Type						
	Active VR (N=31)		Passive VR (N=30)		Control (N=29)		P-value[1]
	Median	(Q1, Q3)	Median	(Q1, Q3)	Median	(Q1, Q3)	
**Observed Pain**							
White	10.0	(0.0,20.0)	0.0	(0.0,20.0)	30.0	(0.0,40.0)	0.3
Non-White	5.0	(0.0,30.0)	0.0	(0.0,50.0)	15.0	(10.0,40.0)	0.9
**Self-reported Overall Pain**							
White	5.0	(0.0,49.0)	8.0	(0.0,70.0)	33.0	(1.0,70.0)	0.4
Non-White	9.0	(0.0,63.0)	10.0	(0.0,60.0)	59.5	(10.0,100)	0.3
**Self-reported Worst Pain**							
White	11.0	(0.0,53.0)	50.0	(0.0,96.0)	34.0	(1.0,80.0)	0.3
Non-White	13.5	(0.0,48.5)	50.0	(0.0,88.0)	70.0	(0.0,100)	0.2

Note: [1] P-value from ANOVA when comparing the means across the three distraction groups

Q1=first quartile (25th percentile), Q3=third quartile (75th percentile)

© 2021 Xiang H et al. *JAMA Network Open.*

eTable 2. Univariate Linear Regression Analysis of Demographic and Burn Characteristics, Pain Medication Prior to Burn Dressing, Child Expectation of VR, and VR Group on Pain Scores, All Children

	**Observed Pain Score**			**Self-reported Overall Pain Score**			**Self-reported Worst Pain Score**		
**Variable**	Parameter estimate	95% CI	P-value	Parameter estimate	95% CI	P-value	Parameter estimate	95% CI	P-value
Age	-2.49	(-4.12,-0.85)	<0.01	-2.81	(-5.20,-0.43)	0.02	-2.51	(-5.02,-0.01)	0.05
Gender	4.00	(-7.41,15.41)	0.49	4.60	(-11.73,20.93)	0.58	4.44	(-12.57,21.46)	0.61
Race(White vs.Non-White)	-2.84	(14.3,8.69)	0.62	-10.93	(-27.3,5.41)	0.19	-8.63	(-25.72,8.47)	0.32
STAQI_CH_score	1.72	(-0.09,3.52)	0.06	3.00	(0.45,5.56)	0.02	2.26	(-0.44,4.96)	0.10
TBSA (%)	3.02	(1.67,4.36)	<0.01	1.78	(-0.38,3.94)	0.10	1.99	(-0.27,4.26)	0.08
Burn_degree	30.93	(10.95,50.91)	<0.01	21.56	(-7.97,51.01)	0.15	25.49	(-5.1,56.07)	0.10
Used pain medication within 6 hours of burn dressing	16.25	(4.41,28.07)	<0.01	20.56	(3.65,37.47)	0.02	20.67	(2.95,38.38)	0.02
Active VR vs. Control	-9.04	(-23.01,4.92)	0.20	-22.20	(-41.80,-2.60)	0.03	-21.34	(-41.69,-0.98)	0.04
Passive VR vs. Control	-4.84	(-18.91,9.23)	0.50	-11.34	(-31.09,8.42)	0.26	-0.89	(-21.41,19.63)	0.93
Child expectation of VR fun for dressing	0.45	(0.17,0.72)	<0.01	0.44	(0.03,0.85)	0.04	0.20	(-0.24,0.64)	0.36
Child expectation of helpfulness of VR	-0.11	(-0.31,0.08)	0.26	-0.13	(-0.41,0.16)	0.38	-0.13	(-0.42,0.17)	0.39

STAQI-CH = State-Trait Anxiety Inventory for Children, TBSA (%)=Total body surface area of burn, 95% CI=95% confidence interval

© 2021 Xiang H et al. *JAMA Network Open.*

eTable 3. Multivariate Linear Regression Analysis of Demographic and Burn Characteristics, Child Expectation of VR, and VR Group on Pain Scores Among Children Who Did Not Use Pain Medication Within 6 Hours of Burn Dressing

	**Observed Pain Score**			**Self-reported Overall Pain Score**			**Self-reported Worst Pain Score**		
**Variable**	Parameter estimate	95% CI	P-value	Parameter estimate	95% CI	P-value	Parameter estimate	95% CI	P-value
Age	-0.00	(-1.68,1.68)	0.99	0.13	(-2.92,3.18)	0.93	-0.81	(-4.26,2.64)	0.64
Gender	1.49	(-9.03,12.02)	0.78	7.42	(-11.63,26.46)	0.44	6.88	(-14.67,28.43)	0.52
Race(White vs.Non-White)	-6.70	(-17.24,3.84)	0.21	-22.05	(-41.12,-2.97)	0.02	-17.07	(-38.65,4.52)	0.12
STAQI_CH_score	-0.20	(-1.81,1.42)	0.81	1.06	(-1.87,3.99)	0.47	0.14	(-3.17,3.46)	0.93
TBSA (%)	2.98	(1.26,4.69)	<0.01	2.25	(-0.86,5.36)	0.15	1.93	(-1.59,5.45)	0.28
Burn_degree	-0.25	(-29.36,28.86)	0.98	8.31	(-44.37,60.98)	0.75	7.30	(-52.32,66.90)	0.81
Active VR vs. Control	-7.32	(-20.43,5.79)	0.27	-26.39	(-50.12,-2.67)	0.03	-17.23	(-44.08,9.61)	0.20
Passive VR vs. Control	-6.03	(-18.74,6.69)	0.35	-24.09	(-47.11,-1.09)	0.04	-6.80	(-32.84,19.24)	0.60
Child expectation of VR fun for dressing	0.33	(0.03,0.63)	0.03	0.50	(-0.03,1.05)	0.07	0.24	(-0.37,0.87)	0.43
Child expectation of helpfulness of VR	-0.13	(-0.33,0.06)	0.17	-0.31	(-0.66,0.05)	0.09	-0.31	(-0.71,0.09)	0.13

STAQI-CH = State-Trait Anxiety Inventory for Children, TBSA (%)=Total body surface area of burn, 95% CI=95% confidence interval

© 2021 Xiang H et al. *JAMA Network Open.*

eTable 4. Multivariate Linear Regression Analysis of Demographic and Burn Characteristics, Child Expectation of VR, and VR Group on Pain Scores Among Children Who Did Use Pain Medication Within 6 Hours of Burn Dressing

	**Observed Pain Score**			**Self-reported Overall Pain Score**			**Self-reported Worst Pain Score**		
**Variable**	Parameter estimate	95% CI	P-value	Parameter estimate	95% CI	P-value	Parameter estimate	95% CI	P-value
Age	-2.72	(-6.22,0.78)	0.12	-5.45	(-10.23,-0.66)	0.03	-4.67	(-10.21,0.86)	0.09
Gender	-11.90	(-34.54,10.74)	0.29	10.92	(-20.03,41.89)	0.47	10.11	(-25.71,45.92)	0.56
Race(White vs.Non-White)	-1.14	(-33.74,31.46)	0.94	6.48	(-38.09,51.06)	0.76	26.72	(-24.85,78.29)	0.29
STAQI_CH_score	2.40	(-2.34,7.14)	0.30	5.46	(-1.01,11.94)	0.09	6.85	(-0.64,14.34)	0.07
TBSA (%)	3.25	(-1.29,7.80)	0.15	-2.43	(-8.65,3.78)	0.43	-0.55	(-7.75,6.63)	0.87
Burn_degree	-14.85	(-60.95,31.24)	0.51	-18.69	(-81.73,44.35)	0.54	-27.28	(-100.21,45.64)	0.44
Active VR vs. Control	22.24	(-6.36,50.85)	0.12	-14.83	(-53.95,24.29)	0.43	-37.85	(-83.10,7.40)	0.09
Passive VR vs. Control	14.93	(-11.47,41.33)	0.25	22.11	(-13.98,58.21)	0.22	21.30	(-20.45,63.06)	0.30
Child expectation of VR need for dressing	0.79	(0.12,1.45)	0.02	-0.36	(-1.27.0.54)	0.41	-0.67	(-1.72,0.38)	0.20
Child expectation of helpfulness of VR	-0.25	(-0.71,0.21)	0.26	0.18	(-0.44,0.80)	0.55	0.45	(-0.26,1.18)	0.20

STAQI-CH = State-Trait Anxiety Inventory for Children, TBSA (%)=Total body surface area of burn, 95% CI=95% confidence interval

© 2021 Xiang H et al. *JAMA Network Open.*

eTable 5. Pediatric Patients With Burns Reported VR Experiences

	Distraction type	
	Active VR (N=31)	Passive VR (N=30)
	Mean (95% CI)	Mean (95% CI)
Fun (0-100, the higher the better)	85.7 (76.6,94.7)	77.3 (65.6,89.0)
Engaging (0-100, the higher the better)	78.9 (68.5,89.3)	72.7 (59.9,85.5)
Realistic (0-100, the higher the better)	73.1 (62.3,83.8)	59.1 (44.7,73.5)

95% CI=95% confidence interval

© 2021 Xiang H et al. *JAMA Network Open.*

eTable 6. Secondary Outcomes of VR Game Experience of Pediatric Patients With Burns

	**Distraction type**		
	**Active VR (N=31)**	**Passive VR (N=30)**	**Control (N=29)**
**Child reported game satisfaction** [1]			
Happy with the VR game	30(97%)	27(90%)	-
Would use it again in future dressing changes	29(94%)	27(90%)	-
**Caregiver reported game satisfaction** [1]			
Happy with the VR game	31(100%)	29(97%)	-
Would use it again in future dressing changes	30(97%)	29(97%)	-
	**Mean (95% CI)**	**Mean (95%CI)**	**Mean (95% CI)**
**Child report – Time spent thinking about pain (0 to 100)** [2,3]	20.3 (8.9,31.8)	34.6 (20.2,49.0)	36.5 (20.8,52.2)
**Clinician reported utility** [1]			
Helpfulness (0-100, higher better)	84.2 (74.5,93.8)	76.9 (65.2,88.7)	-
Easiness (0-100, higher better)	94.8 (91.8,97.8)	96.0 (92.9,99.1)	-
**Length of dressing change (minutes)** [3]	5.2 (4.0,6.4)	4.5 (3.2,5.7)	3.3 (1.9,4.7)
**Simulation sickness** (0-60, lower better)	19.3(17.5,21.1)	19.5(17.6,21.5)	

[1] These questions were not asked for children in the control group

[2] Scale = 0-not at all to 100-very much

[3] Differences in the means by three groups were not statistically significant

95% CI=95% confidence interval

© 2021 Xiang H et al. *JAMA Network Open.*
