# Supplementary material for: Efficacy of Smartphone Active and Passive Virtual Reality Distraction vs Standard Care on Burn Pain Among Pediatric Patients: A Randomized Clinical Trial
Source: JAMA Netw Open. 2021 Jun 21;4(6):e2112082. doi: 10.1001/jamanetworkopen.2021.12082 (PMC8218073; doi:10.1001/jamanetworkopen.2021.12082)
Supplement: Supplement 3. — Data Sharing Statement [file jamanetwopen-e2112082-s003.pdf]

Data Sharing Statement

Xiang. Efficacy of Smartphone Active and Passive Virtual Reality Distraction vs Standard Care on Burn Pain Among Pediatric Patients. *JAMA Netw Open*. Published June 21, 2021. doi:10.1001/jamanetworkopen.2021.12082

Data
Data available: Yes
Data types: Deidentified participant data
How to access data: Patient data are not allowed by our institute (Nationwide Children's Hospital) to share. De-identified data may be available but our legal department must be involved in the negotiation with the requester. Email: xiang.30@osu.edu
When available: With publication

Supporting Documents
Document types: None

Additional Information
Who can access the data: Researchers whose proposed use of the data has been approved, and a data use agreement has been negotiated with legal department of Nationwide Children's Hospital.
Types of analyses: For any purpose or for a specified purpose.
Mechanisms of data availability: After approval of a proposal.
